# Activation of *Hes1* and *Msx1* in Transgenic Mouse Embryonic Stem Cells Increases Differentiation into Neural Crest Derivatives

**DOI:** 10.3390/ijms19124025

**Published:** 2018-12-13

**Authors:** Karla Méndez-Maldonado, Guillermo Vega-López, Sara Caballero-Chacón, Manuel J. Aybar, Iván Velasco

**Affiliations:** 1 Instituto de Fisiología Celular—Neurociencias, Universidad Nacional Autónoma de México, Ciudad Universitaria, Ciudad de México 04510, México; karlam@email.ifc.unam.mx; 2 Laboratorio de Reprogramación Celular del Instituto de Fisiología Celular, UNAM en el Instituto Nacional de Neurología y Neurocirugía “Manuel Velasco Suárez”, Ciudad de México 14269, México; 3 Posgrado en Ciencias Biológicas, Universidad Nacional Autónoma de México; Ciudad Universitaria, Ciudad de México 04510, México; 4 Instituto Superior de Investigaciones Biológicas (INSIBIO, CONICET-UNT), San Miguel de Tucumán T4000ILI, Argentina; gavegalopez@gmail.com; 5 Instituto de Biología “Dr. Francisco D. Barbieri”, Facultad de Bioquímica, Química y Farmacia, Universidad Nacional de Tucumán, San Miguel de Tucumán T4000ILI, Argentina; 6 Facultad de Medicina Veterinaria y Zootecnia, Universidad Nacional Autónoma de México, Ciudad Universitaria, Ciudad de México 04510, México; saracachas@hotmail.com

**Keywords:** β-Tubulin III, smooth muscle actin, collagen 2a, chondrocyte induction, smooth muscle cell induction, stromal-derived inducing activity

## Abstract

The neural crest (NC) comprises a multipotent cell population that produces peripheral neurons, cartilage, and smooth muscle cells, among other phenotypes. The participation of *Hes1* and *Msx1* when expressed in mouse embryonic stem cells (mESCs) undergoing NC differentiation is unexplored. In this work, we generated stable mESCs transfected with constructs encoding chimeric proteins in which the ligand binding domain of glucocorticoid receptor (GR), which is translocated to the nucleus by dexamethasone addition, is fused to either *Hes1* (HGR) or *Msx1* (MGR), as well as double-transgenic cells (HGR+MGR). These lines continued to express pluripotency markers. Upon NC differentiation, all lines exhibited significantly decreased *Sox2* expression and upregulated *Sox9*, *Snai1,* and *Msx1* expression, indicating NC commitment. Dexamethasone was added to induce nuclear translocation of the chimeric proteins. We found that *Collagen IIa* transcripts were increased in MGR cells, whereas coactivation of HGR+MGR caused a significant increase in *Smooth muscle actin* (α-*Sma*) transcripts. Immunostaining showed that activation in HGR+MGR cells induced higher proportions of β-TUBULIN III^+^, α-SMA^+^ and COL2A1^+^ cells. These findings indicate that nuclear translocation of MSX-1, alone or in combination with HES-1, produce chondrocyte-like cells, and simultaneous activation of HES-1 and MSX-1 increases the generation of smooth muscle and neuronal cells.

## 1. Introduction

The neural crest (NC) comprises a transient multipotent cell population that emerges between the forming neural plate and nonneural ectoderm. The NC is a unique and highly specialized group of cells found in all vertebrates. The NC delaminates from the dorsal neural tube following its closure to migrate along different pathways [1,2]. These cells contribute to a wide variety of derivatives, including neural cells of sensory and autonomic ganglia of the peripheral nervous system, the cartilage and bones of the face, melanocytes and smooth muscle cells, as well as other tissues [3,4,5,6]. The induction and specification of NC cells have been studied in different animal models. In avian, amphibian, and fish embryos, NC specification is thought to be initiated during gastrulation or even earlier [7,8,9].

Some of the genes and signals involved in induction of the NC have been identified from studies in chicks, frogs, and zebrafish. Bone morphogenic proteins (BMPs), Fibroblast growth factor (FGF), Wnt, retinoic acid, and Notch participate in this process [10,11,12,13,14,15,16]. Embryonic stem cells (ESCs) derived from mouse and human blastocysts have been extensively employed for studies of developmental processes [6,17,18,19,20]. A recent study in *Xenopus laevis* found that some of the transcription factors associated with the development of the NC are coexpressed with pluripotency factors at the blastula and gastrula stages. In ectodermal explants, high Activin concentrations induced the expression of mesodermal and endodermal markers in the blastula but not gastrula stage. Ectopic expression of either Pax3/Zic1 or Snai2/Wnt8 made gastrula explants competent to produce myogenic differentiation 1 (MyoD) and Endodermin. The authors propose that the NC factors can also be viewed as pluripotency maintenance factors [21]. Recently, in vitro differentiation of human embryonic stem cells demonstrated that Wnt/β-catenin signaling plays an important role in launching early genes that are required for NC development [22].

The importance of other pathways is still being studied: Notch signaling involvement was established through studies in which gain- or loss-of-function of Notch signaling or the Notch effectors, *Hes* genes, were associated with specification, induction or NC migration [23,24,25,26]. However, many experimental approaches are designed in a nonregulated fashion, precluding analysis at different time points during NC induction. For example, mutation of *Hes1* has shown that this gene is essential for neuroblast development in the central nervous system, and therefore, mouse embryos showed abnormalities in neural tube closure, defects in the eyes and ears, as well as craniofacial malformations [27,28].

BMP signaling is relevant during NC differentiation in vivo. Activation of BMP receptors upregulates *Msx1* in the neural fold region. In *Xenopus* multipotent ectodermal tissue (animal caps), a BMP concentration similar to that required to induce the NC increased *Msx1* levels [29]. Recently, a study performed in hESCs demonstrated that BMP signaling is required for NC induction: early inhibition of BMP receptors caused a dramatic inhibition of human NC induction [22]. On the other hand, *Msx1* has been implicated in NC development, since animals with knock-out of this gene die at birth and present multiple craniofacial defects, including cleft palate, as well as a reduction of the jaw and maxilla [30,31]. Similarly, conditional elimination of *Sox9* in the cranial NC, resulted in the absence of cartilages and endochondral bones [32]. Articular cartilage is formed by chondrocytes that express collagens and aggrecan, whereas hypertrophic growth plate chondrocytes undergo apoptosis and provide a template for bone deposition [33].

In *X. laevis* embryos, chimeric versions of *HAIRY2A* (mouse homologue of *Hes1*) and *MSX-1* fused to the ligand binding domain of human glucocorticoid receptor (GR) was used to activate HES-1 and MSX-1 by inducing their nuclear translocation after dexamethasone (Dex) addition. When the chimeric protein contained activation domains, an increase in the NC territories labeled with the markers *SNAI1* and *FOXD3* was observed. Conversely, when a dominant negative mutant of *HAIRY2A* and *MSX-1* was expressed, a decrease in these NC markers was reported. In animal cap assays, stimulation of either of the inducible chimeric proteins (HAIRY2A or MSX-1) with Dex led to upregulation of *Sox2*, a neural plate marker; however, coexpression of *Hairy2a* and *Msx1* produced a decrease in *SOX2* expression and increased the expression of the NC marker *SNAI1*. This indicates that coexpression of both transcription factors induces NC markers in *X. laevis* [16].

The aim of this work was to establish whether HES-1 and MSX-1 participate in the induction/differentiation of the NC using pluripotent mammalian ESCs as a model. To test this hypothesis, we overexpressed inducible forms of HES-1 and MSX-1 proteins in mouse ESCs and evaluated differentiation into NC derivatives, including neural, smooth muscle, and chondrocyte-like cells, after activation of these transcription factors.

## 2. Results

### 2.1. Expression of Hes1 and Msx1 in Wild-type ES Cells in Pluripotent Conditions and after NC Differentiation

**Figure 1:**
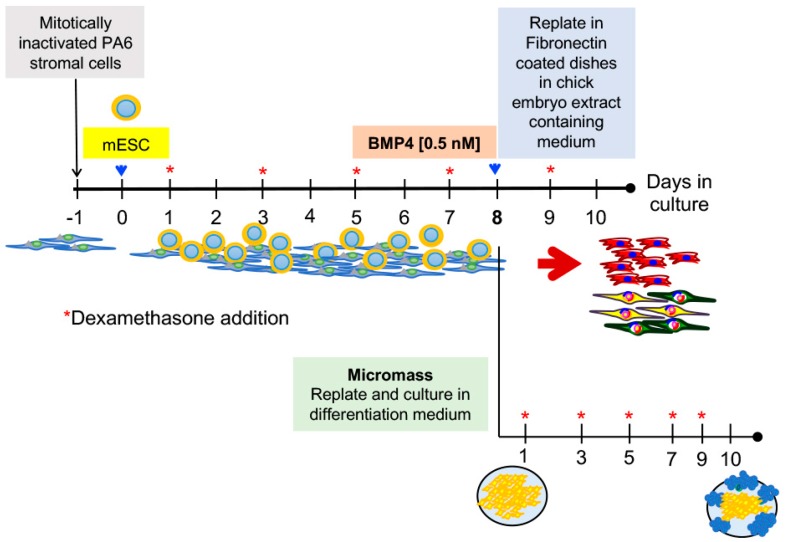
Differentiation of pluripotent cells into neural crest derivatives. The scheme shows the neural crest differentiation protocol. PA6 stromal cells were mitotically inactivated and plated in a monolayer to induce neural differentiation of mouse embryonic stem cells. The addition of BMP4 from day 5 to day 8 promoted the acquisition of a neural crest fate. At day 8, the cells were harvested with Papain and replated in Fibronectin-coated dishes or plated on micromass conditions. After 2 additional days, neurons and smooth muscle cells were detected. The micromasses were stained with Alcian blue after 10 days. Blue arrows indicate replating. The red asterisks indicate the addition of dexamethasone, starting at day one, to promote nuclear entry of the different constructs. The PA6 protocol was modified from [6] and the procedure for micromasses formation was modified from [34].

To analyze the role of *Hes1* and *Msx1* in the differentiation of mESCs into neural crest cells, we used the stromal cell-inducing activity of Pre-adipose 6 (PA6) cells for 5 days [17], followed by the addition of BMP4, which commits cells to differentiate into NC derivatives [6]. Cultures were treated from day 5 to day 8 with 0.5 nM BMP4. At day 8, the cells were harvested by Papain treatment and plated on Fibronectin-coated dishes in neural differentiation medium with chick embryo extract without BMP4, a condition reported to favor differentiation into smooth muscle cells (α-SMA^+^) and neural derivatives (β-TUBULIN III^+^) [6] (Figure 1). Although we did not separate PA6 cells after replating, these cells did not proliferate, and their contribution to the measured parameters was constant for all conditions. At day 8, some cultures were dissociated and replated in micromass conditions to test their chondrogenic potential (Figure 1).

**Figure 2:**
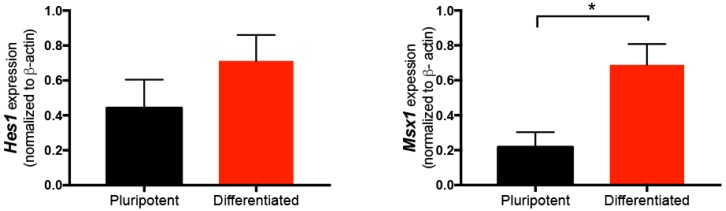
Expression of *Hes1* and *Msx1* in pluripotent conditions and in cells differentiated to NC. Semiquantitative RT-PCR to measure the endogenous transcripts of these two genes in pluripotent W9.5 cells cultured in 2i/LIF, and after 10 days of differentiation in the PA6 protocol. The expression of Hes1 increased non-significantly. On the other hand, we detected a significant increase in Msx1 expression after NC differentiation. mean ± SEM is represented. Statistical analysis was conducted via one-way analysis of variance (ANOVA) followed by Tukey’s multiple comparison test to establish differences. * *p* = 0.032.

To assess the levels of expression of endogenous *Hes1* and *Msx1* in wild-type pluripotent cells, end-point RT-PCR was performed in RNA extracted from cultures grown in 2i/Leukemia inhibitory factor (LIF) without Dex. In W9.5 cells subjected to NC differentiation, we observed a non-significant increase in *Hes1* and a significant rise in *Msx1* (Figure 2). These results indicates that upregulation of *Msx1* is observed after NC induction of mESCs. However, it is not known if a controlled and sustained stimulation, causing nuclear translocation of either or both transcription factors, can influence NC commitemnt during differentiation of pluripotent cells. Therefore, an inducible system might be useful to test this idea.

### 2.2. HES-1-GR and MSX-1-GR Translocate to the Nucleus

**Figure 3:**
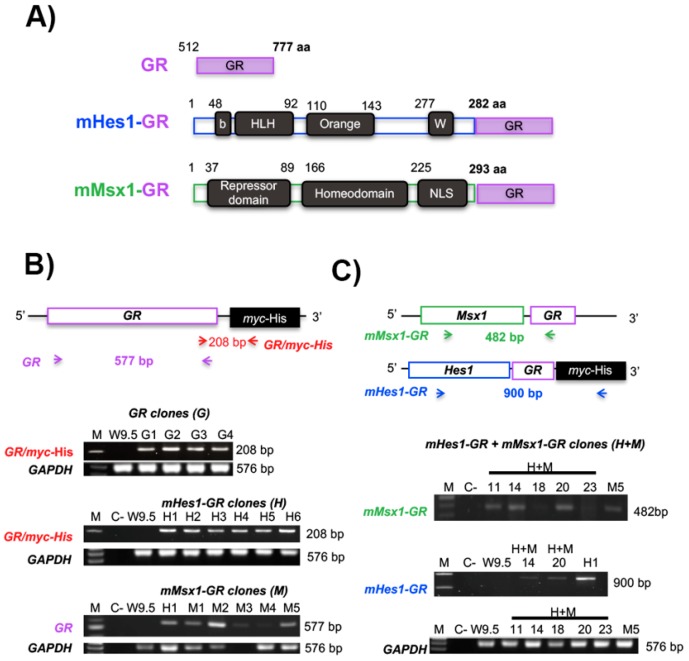
Generation of transgenic mouse embryonic stem cells that express *Hes1* and *Msx1*. (**A**) Scheme of the ligand binding domain of human glucocorticoid receptor (GR) that served as the control. The full-length cDNA of *Hes1* and *Msx1* was cloned in-frame with GR to produce *Hes1*-GR (HGR) or *Msx1*-GR (MGR) vectors. (**B**) After electroporation and selection of mouse embryonic stem cells transfected with the different plasmids, the cells were genotyped via PCR analysis of genomic DNA with the indicated primers, indicated by arrows. The GR fragment was identified with 2 different sets of primers to identify clones, which were designated G. For cells electroporated with the *Msx1*-GR or *Hes1*-GR plasmids, several positive clones were identified and designated H or M, respectively. (**C**) Embryonic stem cells were transfected with both *Hes1*-GR and *Msx1*-GR plasmids. To identify double-positive clones (H+M), the indicated primers were used. Clones 14 and 20 incorporated both constructs. Genomic DNA of W9.5 mouse embryonic stem cells was used as a negative control. (C-) indicates a negative control consisting of water in place of DNA in the PCR reaction.

The use of GR as an inducible system, activated by Dex, allows the timely stimulation of nuclear translocation of transcriptional regulators, to study the effects on cell differentiation. To test if the inducible system was activated by Dex to promote nuclear translocation, we cloned *GR* or *Hes1*-GR (HGR) cDNA sequences into a pcDNA3.1/myc-His vector, which has a 6X-His tag that can be recognized with a specific antibody. We also cloned the *Msx1-GR* (MGR) cDNA sequence into pcDNA3.1-Hygromycin B (Figure 3 and Figure S1). The resulting plasmids were named GR, HGR, and MGR, respectively. These vectors were used to transiently transfect mouse embryo fibloblast (NIH-3T3) cells to evaluate nuclear translocation after Dex addition more easily than in mESCs, which have a very reduced cytoplasm. We assessed different concentrations of Dex (10, 50 and 100 μM) to obtain maximal nuclear translocation of each construct, and we decided to stimulate with the lowest tested concentration that caused a significant translocation effect. We measured the nuclear translocation using ImageJ software. In HGR cells, we quantified the fluorescence using an equation to obtain the Corrected Cell Fluorescence (CCF) in the nucleus (Figure S2A). We observed an increase in the nuclear CCF after inducing nuclear translocation with Dex. In the case of MGR, we counted cells that had an exclusive nuclear signal after immunostaining and quantified the percentage of MSX-1 cells with positive labeling (Figure S2B). After confirming that this system worked in mouse cells, we generated stable transgenic mESC lines.

### 2.3. Generation of Transgenic Stable mESC Lines

We transfected W9.5 mESCs with the GR, HGR, MGR, or both the HGR and MGR plasmids by electroporation, followed by antibiotic selection with 300 μg/mL G418 (GR and HGR), 200 μg/mL Hygromycin B (MGR) or both antibiotics for HGR+MGR for 10 days. The cells were maintained with 100 μg/mL G418 or 110 μg/mL Hygromycin B for 11 more days to obtain several colonies, which were picked and expanded in Knock Out-Dubelco’s Modified Eagle’s Medium (KO-DMEM) with LIF and 2i. We obtained genomic DNA from these clones to identify the transgenes using specific primers for GR/myc-His (208 bp), GR (577 bp), MGR (482 bp) or HGR (900 bp), as presented in the scheme shown in Figure 3B,C. *Gapdh* was also detected (576 bp). After genotyping, we identified several clones with the GR (four clones), HGR (six clones), MGR (five clones) or HGR+MGR (two clones) constructs (Figure 3B,C). The identified positive clones were selected for differentiation based on the best morphology of the pluripotent colonies. 

**Figure 4:**
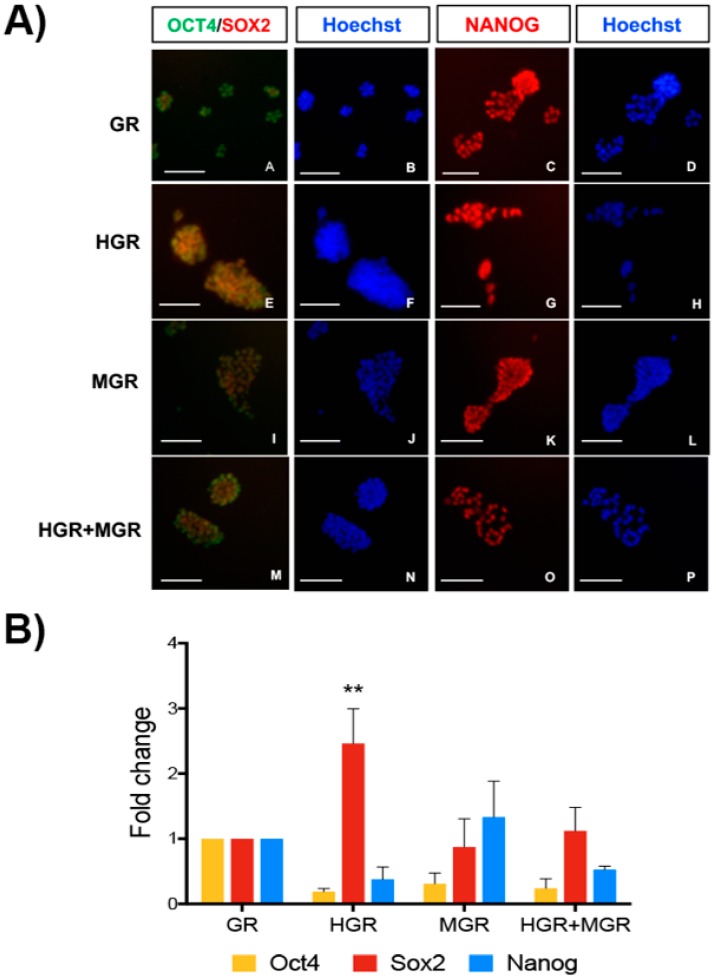
Transgenic mESCs remain pluripotent. (**A**) We evaluated the expression of OCT4, SOX2 and NANOG in the transgenic cells via immunofluorescence and observed that the selected clones continued to express these pluripotent factors. Scale bars = 100 μm. (**B**) The expression of pluripotency factors was evaluated by RT-qPCR in all transgenic cell lines. Two-way ANOVA followed by Tukey’s test indicated only a significant difference in Sox2 expression for the HGR clone compared with the control GR clone after comparison of all cell lines. ** *p* < 0.0025.

To further assess whether these transgenes affected pluripotency markers, we performed immunostaining for OCT4, SOX2, and NANOG in the parental cell line W9.5 and the selected clonal lines (Figure 4A), and we observed that the transgenic lines were positive for these markers. Consistently, RT-qPCR detection of *Oct4*, *Sox2* and *Nanog* showed similar levels in all transgenic cell lines, except for a significant increase in *Sox2* in HGR cells (Figure 4B). This increase does not affect the pluripotent state, since mESCs can express high levels of *Nanog* [35] and HGR cells continue to express *Oct4* and *Sox2* at the mRNA and protein levels; these 3 transcription factors conform the core positive autoregulatory network of pluripotency [36].

### 2.4. Expression of the Human Glucocorticoid Receptor, Hes1 and Msx1 are Similar in the Transgenic Cell Lines before Differentiation.

**Figure 5:**
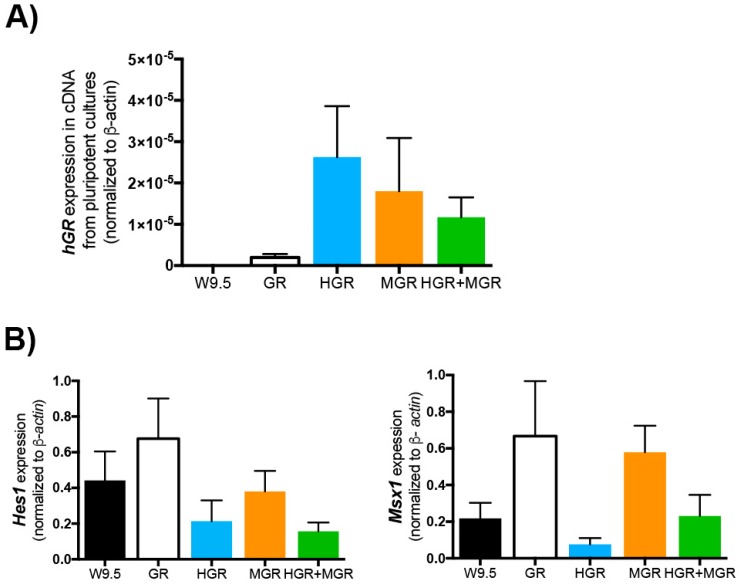
RT-PCR to detect the transgenic human glucocorticoid receptor and the endogenous *Hes1* and *Msx1* in pluripotent wild-type and transgenic lines. RNA was obtained from W9.5, GR, HGR, MGR, and HGR+MGR cells growing in 2i/LIF. Retrotranscription was made to prepare cDNA, which was amplified by qPCR (**A**) with the primers listed in Methods. Wild-type W9.5 mESCs did not express the transgene. The expression of the transgenic sequence present in all plasmids was normalized to β-actin. (**B**) Semiquantitative RT-PCR was used to measure transcripts of these two genes in pluripotent W9.5 (non-transgenic) and transgenic (GR, HGR, MGR, and HGR+MGR) cells, cultured in 2i/LIF. mean ± SEM is represented. Statistical analysis was conducted via one-way ANOVA followed by Tukey’s multiple comparison test to establish differences. No significant differences were found between the four transgenic cell lines in 3–4 independent experiments.

Since the integration of plasmids in mESCs is randomized, we tested the expression level of the human GR, which is included in all the transgenic constructs, in cDNA prepared from cultures growing with 2i/LIF, using wild-type W9.5 mESCs as a negative control. Quantitative PCR in W9.5 cells did not result in amplification as expected. All transgenic lines express the transgene. We did not find significant differences, suggesting that all lines express comparable levels of transgenes (Figure 5A). Additionally, to compare the endogenous levels of expression of *Hes1* and *Msx1* in wild-type and transgenic pluripotent cells, end-point PCR was performed in cDNA prepared from cultures grown in 2i/LIF without Dex. No significant differences were detected in the expression of these two genes in undifferentiated conditions (Figure 5B).

### 2.5. Neural and NC Marker Expression in Transgenic Pluripotent and Differentiated Cells

**Figure 6:**
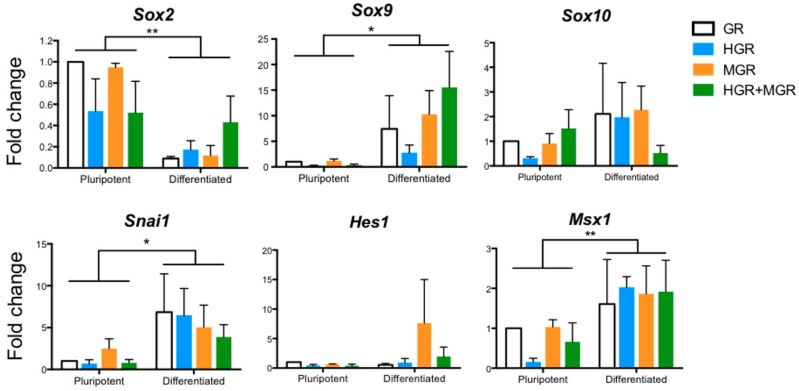
Induction of neural crest markers in differentiated cells in the absence of dexamethasone. The transgenic cells were differentiated into neural crest derivatives over 10 days. The expression of several markers was measured using end point RT-PCR. The expression of each gene was normalized to β-*actin* expression, and the fold change was determined relative to the levels found in pluripotent GR cells. mean ± SEM. Statistical analysis was conducted via two-way ANOVA followed by Tukey’s multiple comparison test to establish differences between pluripotent and differentiated cells from 3 independent experiments. * *p* < 0.01; ** *p* < 0.001. No differences were found between the four cell lines when compared at in the pluripotent state or after differentiated conditions, indicating that the significant differences are caused by the differentiation protocol.

After 10 days of differentiation under control conditions, we extracted RNA and performed RT-PCR to analyze the expression of different NC markers (Figure 6). The expression of each gene was normalized to *β-actin* levels and is presented as fold change with respect to expression in control pluripotent cells (GR). In this assay, we analyzed the expression of endogenous *Msx1* and *Hes1*, as well as *Sox2* and NC markers (*Sox9*, *Sox10*, and *Snai1*) after NC differentiation. We observed significant differences when mESCs were subjected to differentiation into NC derivatives. *Sox2* expression decreased, whereas *Sox9*, *Snai1*, and *Msx1* expression was augmented in all cell lines relative to pluripotent GR cells. No significant differences were found in *Sox10* and *Hes1* between the four cell lines under pluripotent or differentiated conditions. These molecular changes are consistent with the transcriptional response observed after NC differentiation of ESCs [19,22].

**Figure 7:**
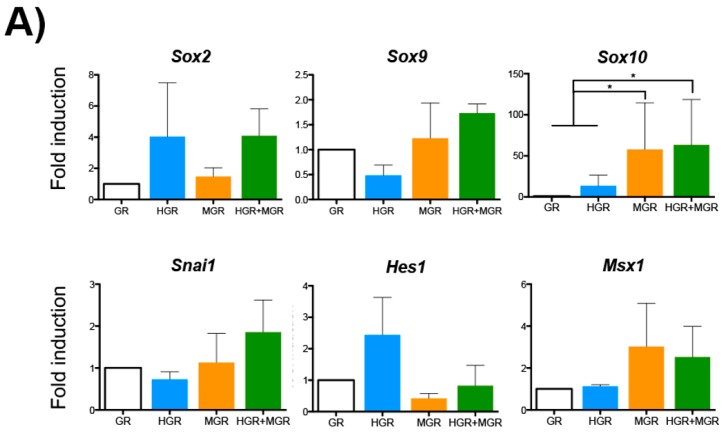

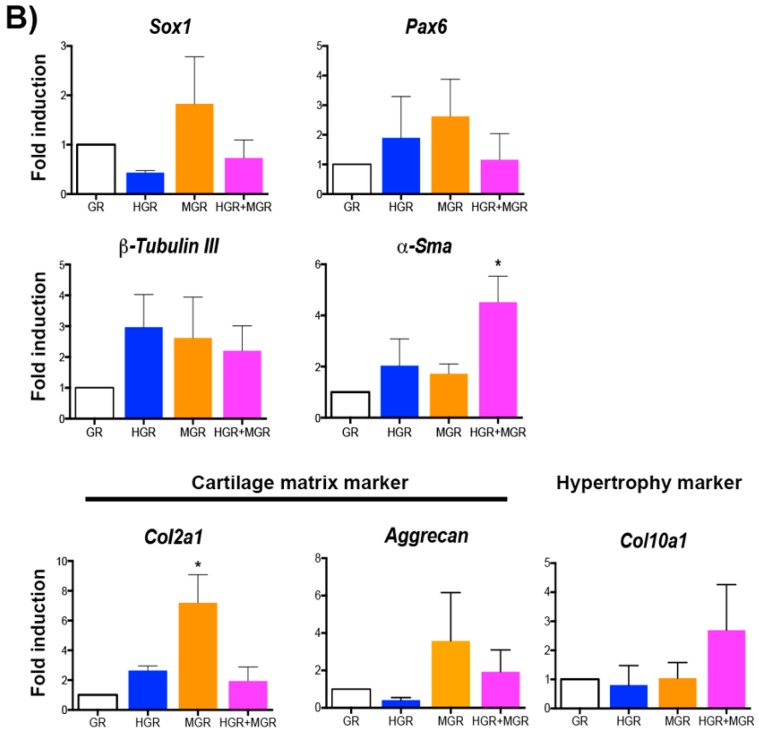
Activation of nuclear translocation of chimeric proteins during differentiation of mESCs into NC derivatives increased the *Col2a1* transcripts in MGR cells and α-*Sma* transcripts in double transgenic cells. Transgenic mESCs were differentiated into neural crest derivatives and stimulated with 10 μM Dex to promote nuclear translocation of the transgenic proteins from day 1. Analyses were conducted at day 10 of differentiation. (**A**) End point RT-PCR showed significant increases in *Sox10* expression in MGR and HGR+MGR cells relative to GR and HGR cells after stimulation of nuclear translocation. Please note that the primers for *Hes1* and *Msx1* detect the endogenous forms and not the transgenes (**B**) Quantification of the neural and neural crest differentiation markers via RT followed by qPCR. The fold induction was relative to the levels in GR cells. The data for each transcript were normalized to β-actin levels. mean ± SEM. Statistical analysis of all conditions was conducted via ANOVA followed by Tukey’s multiple comparison test from 3–4 independent experiments. The marked significant differences are relative to GR, unless indicated otherwise. * *p* < 0.05.

To evaluate the role of HES-1 and MSX-1 activation, Dex was added from day 1 of differentiation. RNA was obtained at day 10 of differentiation to evaluate the same genes using semiquantitative RT-PCR. It is noteworthy that the primers for *Hes1* and *Msx1* detect the endogenous forms and not the transgenes. We only observed significant increases in Sox10 expression in MGR and HGR+MGR cells relative to the control GR and HGR cells after stimulation of nuclear translocation (Figure 7A). Evaluation of NC markers via RT-qPCR revealed that in Dex-treated double-transgenic cells, the activation of HES-1 and MSX-1 nuclear translocation increased α-*Sma* transcripts, suggesting increased differentiation to smooth muscle cells, one type of NC derivative (Figure 7B). In contrast, the levels of other genes related to neural differentiation (*Pax6*, *Sox1*, and *β-Tubulin III*) did not change. Analysis of additional NC markers such as Type II Collagen gene (*Col2a1*) expression, characteristic of chondrocytes, was increased in MGR cells, but other chondrocytic markers (*Aggrecan* and *Col10a1*) were unaffected (Figure 7B). 

### 2.6. Activation of HES-1 and MSX-1 Increased the Number of β-TUBULIN III- and α-SMA-positive Cells

**Figure 8:**
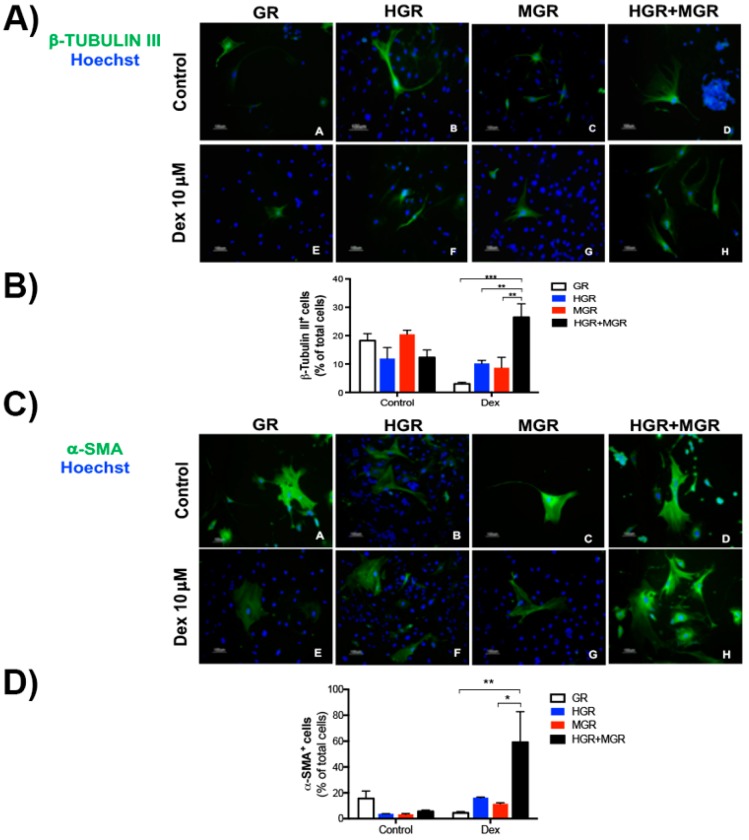
Activation of HGR and MGR nuclear translocation in double transgenic cells increases the number of differentiated β-TUBULIN III- and α-SMA-positive cells. Cells were differentiated under control conditions or with 10 μM Dex for 10 days and immuno-stained with anti-β-TUBULIN III antibody (**A**). (**B**) Quantification of the number of β-TUBULIN III^+^ cells. Double-transgenic cells produced a higher proportion of neurons compared with GR, MGR, or HGR cells. (**C**) Immunostaining with anti-α-SMA antibody after differentiation under control and dexamethasone-added conditions. (**D**) Quantification of large α-SMA-positive cells with a filamentous aspect. Double-transgenic cells produced a higher proportion of smooth muscle cells compared with GR and MGR cells. Experiments were performed in triplicate; pictures were taken in at least ten fields from three independent experiments. The mean ± SEM is presented. Statistical analysis of all conditions was conducted with ANOVA followed by Tukey’s multiple comparison test and only the significant differences are indicated in the graph. * *p* < 0.05, ** *p* < 0.01, and *** *p* < 0.001.

Transgenic mESC lines differentiated using the NC protocol with PA6 stromal cells were fixed at day 10 to perform immunofluorescence staining with specific antibodies. We detected the expression of *β-Tubulin III* in cells that were small and exhibited a multipolar morphology under all conditions (Figure 8A); quantification of immuno-positive cells showed that the number of β-TUBULIN III-positive cells significantly increased when the nuclear translocation of HES-1 and MSX-1 was induced with Dex (Figure 8B). Since real-time PCR indicated that nuclear translocation in double-transgenic cells upregulated Smooth muscle actin expression, antibodies for α-SMA were used to examine differentiated cells. The control and treatment conditions produced large, filamentous and polyhedral α-SMA^+^ cells (Figure 8C); however, quantification of the number of cells positive for this marker showed a significant increase when HGR+MGR cells were activated (Figure 8D).

### 2.7. Activation of MGR and Double-transgenic Cells Increases Chondrocyte Differentiation.

**Figure 9:**
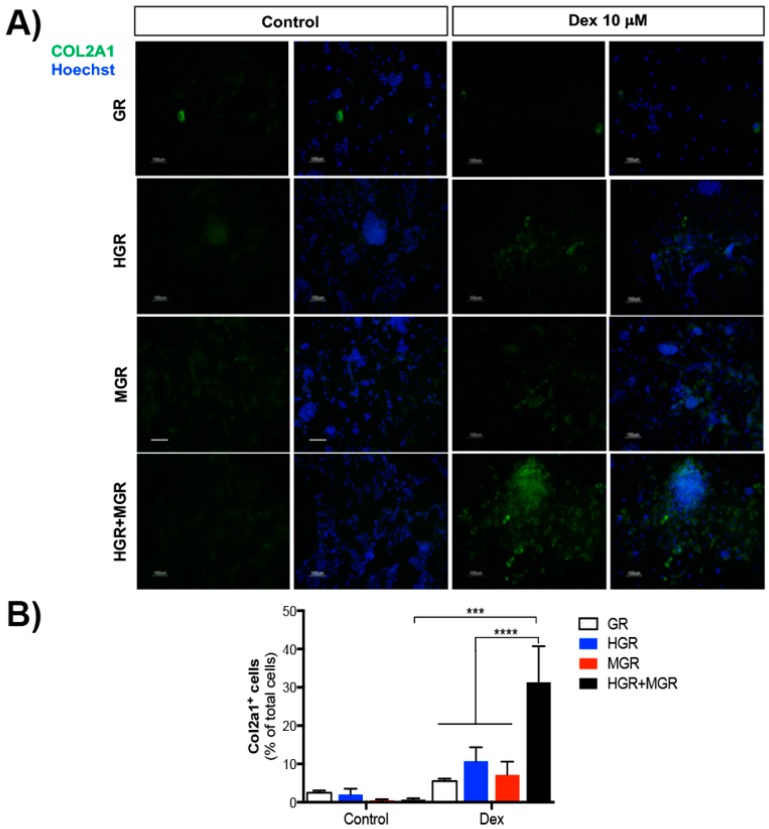
Activation of HGR and MGR nuclear translocation in double transgenic cells increases the number of differentiated COL2A1-positive cells. Cells were differentiated under control conditions or with 10 μM Dex for 10 days and immuno-stained with anti-COL2A1 antibody (**A**). (**B**) Quantification of the number of COL2A1^+^ cells. Double-transgenic cells produced a higher proportion of chondrocytes compared with stimulated GR, MGR, or HGR cells as well as unstimulated double transgenic cells. The mean ± SEM from 3 independent experiments is presented. Statistical analysis of all conditions was conducted with ANOVA followed by Tukey’s multiple comparison test and only the significant differences are indicated in the graph. The scale bars represent 100 μm *** *p* < 0.001, and **** *p* < 0.0001.

**Figure 10:**
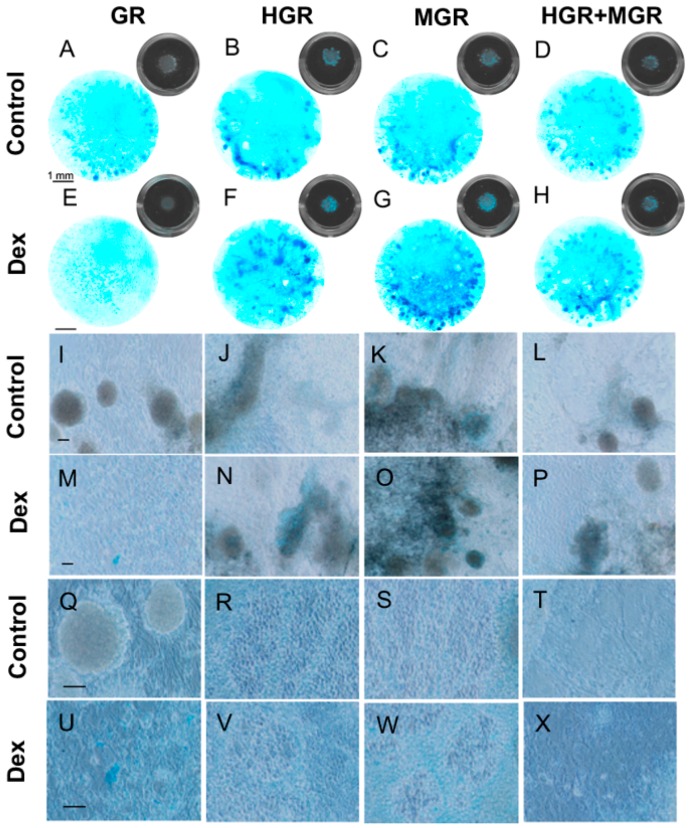
Activation of MSX-1 increases the proportion of Alcian blue-positive cells in micromass cultures. Cells were differentiated on PA6 cells for 8 days and replated as micromass aggregates for 10 additional days. In control conditions (**A**–**D**, **I**–**L** and **Q**–**T**), all cell lines presented a few Alcian blue-positive aggregates. The proportion of blue cells clearly increased after Dex addition (**E**–**H**, **M**–**P** and **U**–**X**), especially in MGR cells (**G**,**O**,**W**). Blue aggregates of cobblestone-like cells suggest the presence of chondrocytes (**R**,**S**,**W**), with larger cells that might correspond to hypertrophic chondrocytes (**T**,**X**) in the HGR + MGR cells. The black inset (**A**–**H**) show the well of the 24-well plate with the micromass growing on the center. The scale bars represent 1 mm (**A**–**H**), 500 μm (**I**–**P**) and 100 μm (**Q**–**X**).

Immunostaining of cells stimulated to differentiate to NC for 10 days, with or without Dex, revealed a significant increase in COL2A1-positive cells in HGR+MGR transgenic cells, after activation of both transcription factors (Figure 9). To analyze if chondrogenic differentiation can proceed further, cells were differentiated on PA6 cells for 8 days and replated as micromass culures in differentiation medium without chick embryo extract (Figure 1) [34]. These cultures were subjected to Alcian blue staining to reveal proteoglycan characteristic of extracellular matrix produced by chondrocytes. After 3 days, no positive cells were detected, regardless of Dex addition. After 10 days all cell lines showed a few blue cells in the absence of Dex, and activation of MSX-1 clearly increased the proportion of Alcian blue-positive cells. Furthermore, blue aggregates of cobblestone-like cells were more abundant in MGR cells stimulated with Dex (Figure 10).

## 3. Discussion

In this study, we used human GR as an inducible system. We verified that *Msx1* and *Hes1* fused to *GR* responded to Dex addition by nuclear translocation. Stable transgenic mESC lines containing *GR*, *HGR*, *MGR*, or *HGR*+*MGR* were generated, maintained the expression of pluripotency-associated markers, and responded to a differentiation protocol for NC induction. After differentiation of the transgenic cell lines to obtain neural crest derivatives and treatment with Dex to induce nuclear translocation of the transcription factors during the differentiation progress, we found the following: 

(1) Nuclear translocation of HES-1 and MSX-1 in double-transgenic cells significantly increased expression of *Sox10*, as well as differentiation to neurons and smooth muscle cells by augmenting the number of cells positive for β-TUBULIN III and Smooth Muscle Actin, respectively.

(2) Transgenic lines activated to translocate MSX-1 or HES1+MSX1 showed increased chondrogenic differentiation assessed by expression of COL2A1, immunostaining for COL2A1 or Alcian blue staining. 

Hes basic Helix-Loop-Helix (bHLH) repressors encode nuclear proteins that repress transcription, and they have three conserved regions that confer transcriptional functions: the bHLH (DNA binding and region for dimerization), Orange (selection of bHLH heterodimer partners) and WRPW (represses transcription) domains [37,38,39]. *Hes* genes play an essential role in the development of many organs by maintaining progenitor cells and regulating binary cell fate decisions. During late neural development, HES-1 regulates an astrocyte versus oligodendrocyte fate choice in glial restricted precursors [40]. On the other hand, *Hes* genes have a role in cell cycle [28]. HES-1 has an important function in self-renewal and cell survival in multipotent neural cells in the central nervous system [41]. This transcription factor has an important role in neural development, but the role in NC induction in mammals remains unclear. We did not observe induction of further NC differentiation in HGR cells after Hes1 nuclear translocation. 

It has been reported that HES-1 increases the promoter activity of α-*Sma* in hepatic stellate cells [42], although previous studies reported that activation of Hes1 by juxtacrine Notch signaling did not increase α-SMA protein levels in the same cell type [43]. In contrast, high levels of HES-1 inhibit the ability of MyoD to convert 10T1/2 mouse embryonic fibroblasts to muscle cells [44]. Our results show that neither activated HES-1 nor MSX-1 increased α-*Sma* transcript or protein levels. However, the coactivation in HGR+MGR cells showed a significant effect, increasing the number of α-SMA-positive cells and α-*Sma* mRNA levels. Interestingly, a previous paper showed that upon differentiation in the presence of TGF-β human iPSC-NC stem cells produced a Smooth Muscle lineage that expressed α-*Sma*, [45]. Thus, the regulation of expression of α-*Sma* seems to be dependent on the partners of Hes1 and/or the cell type.

On the other hand, there is extensive evidence that MSX-1 transcription factors have an important role in proliferation, differentiation, and NC induction during embryonic development. *Msx1* and *Msx2* are specifically required for osteogenesis in the cranial NC lineage. *Msx1* is strongly expressed in these cranial cells and plays a critical role in regulating epithelial to mesenchymal transition (EMT) during organogenesis [46,47]. *Msx1* and *Msx2* function redundantly in multiple tissues and organs during vertebrate development, including the heart. *Msx1*-/- *Msx2*-/- double-knockout mice have profound defects in the cardiac outflow tract. These defects were associated with excessive proliferation of cardiac NC cells [31]. A study with *Msx1*/*Msx2* double-null mice investigated the effect of eliminating these genes on atrioventricular cushion and valve development. The researchers demonstrated that mutant mice had impaired EMT, resulting in hypoplastic cushions and malformed valves. Interestingly, α-*Sma* is expressed prior to EMT in smooth muscle cell precursors; in double-knockout animals, decreased expression of α-*Sma* is reported, which can contribute to defective atrioventricular cushion formation [48]. The NC is one of the major sources of smooth cell muscle cells in blood vessels (i.e., the aorta, ductus arteriosus, common carotid, innominate and right subclavian arteries) [49,50,51,52]. Taken together, these results suggest that simultaneous activation of MSX-1 and HES-1 participate in the regulation of α-*Sma* expression. 

SOX proteins are candidates for participation in NC specification. They are involved in several processes during embryogenesis. Based on the amino acid sequences of the HMG domain, SOX proteins can be divided into ten subgroups. Subgroup E consists of three members (SOX8, SOX9 and SOX10) that are expressed in several developing tissues, including the NC [53]. *Sox9* is an early marker of prospective NC cells, preceding markers of migratory cells. *Sox9* is the first to be expressed, overlapping with the expression of BMP4 and *Snai2* [48,54]. Sox10 is expressed shortly after SNAI2 and is required for the early survival and migration of NC cells, and later on for differentiation of Schwann cells and melanocytes. *Sox10* expression continues in glia development but is downregulated in other NC lineages [55]. 

In vivo, neural crest cells constitute a transient population that arise from the ectoderm and migrates away and differentiate into many derivatives. Early work described that multipotent NC stem cells can be isolated from the rat sciatic nerve [56] or from postmigratory cells of the mouse first branchial arch [57]; such cells can differentiate in vitro to neurons, osteoblasts, myofibroblast (positive for α-SMA), and Schwann cells, even at clonal assays. This suggests that a rare niche of NC stem cells might be present in vivo, but cultured cells can present a different behavior in terms of progenitor gene expression and terminal differentiation. Supporting this notion, multipotent “crestospheres” from premigratory chick embryos express the NC genes *Sox9*, *Ets1*, *Bmp4*, and *Snai2*, as well as the neural marker *Pax6* [58]. These NC cells differentiated to neurons, melanocytes, smooth muscle cells, and chondrocytes, both in vitro and after transplantation in the head mesenchyme of chicks. Induction of the premigratory NC population from neural rosettes derived from human ESCs produced crestospheres that differentiated to neurons, melanoblasts, smooth muscle cells, and chondrocytes, but in conjunction with neurospheres positive for Sox2. These results highlight the differences observed when studying NC progenitors in vivo and those differentiated in vitro from ESCs. We observed significant increases in *Sox9* and *Snai1* expression after NC induction under control conditions, indicating proper NC induction of mESCs. Furthermore, in MGR and HGR+MGR cells stimulated with Dex, significant upregulation of *Sox10* was observed. This suggests that the NC progenitor state is enriched under these conditions. However, enhanced differentiation to neurons, smooth muscle, and chondrocyte-like cells was also observed, which might reflect cell populations at different stages of differentiation to NC derivatives in these cultures. To our knowledge, this is the first report that indicates that activation of HES-1 and MSX-1 in mESCs produce higher proportions of neurons, smooth muscle cells, and chondrocytes, derivatives obtained also from the differentiation of NC stem/progenitor cells, although the molecular mechanisms remain to be identified.

Previously reported evidence demonstrated that morphogens, such as BMPs, together with the transcription factor SOX9, are crucial for chondrogenesis. Wnt1-driven *Sox9* conditional knockout present defects in cartilage production in cranial structures [32]. SOX9 is the transcription factor that activates Collagen IIa (*Col2a1*) [59]. On the other hand, the *Col2a1* gene can also be controlled by Barx2, a homeodomain transcription factor [60]. Chondrocytes can be articular or growth plate (hypertrophic): the former give rise to cartilage and the latter provide a template on which new bone is formed, substituting hypertrophic cells. Hypertrophic chondrocytes express *Col10a1* [61].

Activation of *Msx* genes by BMP signaling can promote cartilage development. For example, exogenous BMP-7, applied to lateral mandibular region at an early stage of chick development, causes ectopic expression of *Msx1* and inhibited the formation of Meckel´s cartilage. In contrast, BMP-7 addition at later stages up-regulated *Msx1*, but the final result was increased chondrogenesis [62]. Another paper where chick embryos were transduced with BMP receptors in craniofacial structures, showed that the activation of these receptors upregulated *Msx1* and *Msx2* transcripts in regions that normally do not express these genes, causing overgrowth of cartilage elements in the head; the authors conclude that the activation of BMP receptors promotes chondrogenesis [63].

We found that MSX-1 activation in MGR cells induces upregulation of *Col2a1* transcription, as well as an increase in Alcian blue staining of micromass cultures, an effect not previously reported, but that might have interesting implications for cartilage development. In fact, the expression of *Col2a1* is a sign of chondrocyte maturation for cells differentiated from mESCs, and was observed when using a different protocol, involving embryoid bodies and cell aggregation, to produce chondrocytes [33]. Craft and coworkers used different induction protocols for the production of articular o hypertrophic chondrocytes. They described differences in morphology and Collagen2a1 protein levels: hypertrophic cells are larger and had higher levels of Collagen 2, compared to articular cells [33]. Our results indicated that in MGR cells stimulated with Dex chondrogenesis occurred by the expression of *Sox9* and *Col2a1*. Furthermore, stimulated MGR cells replated in micromass conditions showed stronger staining for the extracellular matrix components detected by Alcian blue. Interestingly, HGR+MGR cells stimulated with Dex showed increased proportions of COLLAGEN 2A1-positive cells, larger chondrocyte-like cells and an increased, albeit non-significant, expression of the hypertrophic marker Col10a1, suggesting that simultaneous activation of HES-1 and MSX-1 promotes a growth plate chondrocyte-like phenotype.

## 4. Materials and Methods

### 4.1. Cell Culture

#### 4.1.1. NIH-3T3 Cells

The NIH-3T3 mouse embryonic fibroblast cell line (ATCC^®^CRL-1658^TM^) was cultured in Dulbecco’s Modified Eagle’s Medium (DMEM) containing 10% fetal bovine serum (FBS), 2 mM Glutamax (GIBCO^®^, Waltham, MA, USA), and 50 U/mL penicillin-streptomycin (Thermo Fisher Scientific, Waltham, MA, USA) in gelatin-coated dishes at 37 °C with 5% CO_2_. 

#### 4.1.2. ESCs

Mouse ESCs of the W9.5 line (gift of Diana Escalante-Alcalde Lab) were grown in Knock-Out Dulbecco’s Modified Eagle Medium high-glucose (KO-DMEM) containing 15% FBS, Leukemia Inhibitory Factor (1000 U/mL), 0.1 mM nonessential amino acids, 2 mM Glutamax (GIBCO^®^, Waltham, MA, USA), 55 μM 2-mercaptoethanol, and 50 U/mL penicillin-streptomycin (Thermo Fisher Scientific, Waltham, MA, USA). To maintain the pluripotent state, ESCs were grown with 2i: the GSK-3β inhibitor CHIR99021 (3 μM, EMD Millipore Co., Hayward, CA, USA) and the Extracellular signal-regulated kinases (ERK) inhibitor PD0325901 (1 μM, Sigma-Aldrich, Milwaukee, WI, USA) [64].

#### 4.1.3. PA6 Cells

PA6 stromal cells (RIKEN BioResource Center) were used for their stromal cell-derived neural inducing activity. These cells were cultured in Minimum Essential Medium (α-MEM) containing 10% FBS and 50 U/mL penicillin-streptomycin (Thermo Fisher Scientific, Waltham, MA, USA). PA6 cells were cultured in gelatin-coated plates at 37 °C in 5% CO_2_. To avoid nutrient depletion of the culture medium, PA6 cells were inactivated mitotically with 10 μg/mL Mitomycin C for 2 h at 37 °C in 5% CO_2_. After 2 h, the cells were washed with PBS, harvested and maintained in culture with α-MEM until used for differentiation of mESCs.

### 4.2. Generation of cDNA Constructs and the Inducible System

The development of hormone-inducible proteins allows accurate control of the time at which they act [65,66]. To temporally control overexpression of *Hes1* and *Msx1* and coexpression of *Hes1-Msx1*, the ligand binding domain of the human GR was amplified with the GR-Fw (SacI) 5′-GAGCTCcacaagaaacctctgaaaatcctggtaac-3′ and GR-Rev (XhoI) 5′-CTCGAGctgattggtgatgatttcagc-3′ [15] and cloned upstream in frame (into SacI and XhoI sites) with the coding sequence of a myc-His construct. This control plasmid only expresses encoded GR and the myc-His tag. The Mus musculus Hes1 ORF and *Msx1* ORF sequences were high-fidelity PCR amplified from the full-length cDNA clone ID 6478994 (cat. # MMM1013-9200434, Open BioSystems Inc., Dharmacon, Lafayette, CO, USA) using the following primers: *mHes1*-Fw (HindIII) 5′-AAGCTTatgccagctgatataatggagaaa-3′; *mHes1*-Rev (BamHI) 5′-GGATCCagttccgccacggtctcca′; *mMsx1*-Fw (HindIII) 5′-AAGCTT atgacttctttgccactcggtg-3′; *mMsx1*-Rev (BamHI) 5′-GGATCC aagtcaggtggtacatgctgtagcc-3′) and ligated into a pCR-Topo-TA vector. The amplified fragment encoded 284 amino acid residues of *Hes1*, without the stop codon, and included its three structural domains, bHLH, Orange and WRPW. *Msx1* (cDNA clone ID 4923403, cat. # MMM1013-202766935) contained the repressor domain, homeodomain (DNA binding) and nuclear localization signal (NLS). The pTopo-*Hes1* vector and pTopo-*Msx1* vectors were cut and then directionally subcloned into HindIII and BamHI sites in the GR vector to produce the *Hes1*-GR-pcDNA3.1-myc-His construct and *Msx1*-GR was subcloned into pcDNA3.1-Hygro, as shown in Table 1. DH5α cells were used to expand the plasmid, and the colonies were selected with 100 µg/mL ampicillin. Plasmid DNA was obtained and sequenced to verify each construct.

### 4.3. Nuclear Translocation after Dexamethasone Stimulation

NIH-3T3 cells were used to express the hGR-pcDNA3.1-myc-His (GR), Hes1-hGR-pcDNA3.1-myc-His (HGR) or Msx1-hGR-pcDNA3.1-Hygro (MGR) constructs. Cells were transfected using Lipofectamine 2000 (Invitrogen, Carlsbad, CA, USA) according to the manufacturer’s instructions. Twenty-four hours before transfection, NIH-3T3 cells were cultured in DMEM without phenol red supplemented with 10% charcoal-stripped FBS to avoid activation of GR before Dex addition. Forty-eight hours after transfection, the cells were treated with 10 μM dexamethasone (Dex; D2915, Sigm -Aldrich, Milwaukee, WI, USA) to evaluate nuclear translocation of the constructs. We stimulated the cells with Dex for 24 h, assessed nuclear translocation using specific antibodies and analyzed the staining using ImageJ program (1.51 p 22). In brief, we selected, using the freeform drawing tool, the nucleus of each cell stained with the specific antibody recognizing the 6X-His tag to identify GR and HGR translocation or anti-MSX-1/2 antibodies for MGR. After selection of the area of interest, we chose a region next to the cell that had no fluorescence, defined this as the background level, and subtracted the contribution of the background in the selected area. We then quantified the integrated density and mean gray value and used these data to calculate the corrected cell fluorescence (CCF) in the nucleus using the equation CCF = Integrated density − (area of selected cell × mean fluorescence of background readings). 

### 4.4. Generation of Stable mESC Lines

Two-and-a-half million mESCs were electroporated separately with 3 μg of the GR, HGR, or MGR plasmids, or with both the HGR and MGR plasmids using the Nucleofector system (Lonza, Basilea, Suiza). After electroporation, cells were cultured in KO-DMEM with LIF and 2i. Two days after electroporation, mESCs were treated with 200 μg/mL G418 (Sigma-Aldrich, Milwaukee, WI, USA) or 300 μg/mL Hygromycin B (Sigma-Aldrich, Milwaukee, WI, USA) for 10 days to select resistant clones. Clones were expanded and maintained with 100 μg/mL G418 or 110 μg/mL Hygromycin B in the presence of LIF and 2i before differentiation. 

### 4.5. Genotyping of the Resistant Clones

To detect integration of specific cDNA into the mouse genome, we obtained genomic DNA and evaluated the presence of human *GR, HGR* or *MGR* via end-point PCR using specific primers (all listed 5′-3′). For GR: Forward (Fw)-gttggaggttattgaacctgaag and Reverse (Rev)-ttcatgcatagaatccaagag; GR/myc-His: Fw-ctgacaaaactcttggattctatgc and Rev-tgaatatgcataccggtcatcatc; m*Msx1*-GR (MGR): Fw-cctacgcaagcacaagacca and Rev-ggtaggggtgagttgtggta; *mHes1*-GR (HGR): Fw-tctacaccagcaacagtgg and Rev-tgaatatgcataccggtcatcatc. The amount of DNA was assessed with the following specific primers for *Gapdh* (NM_001289726.1): Fw-atcaccatcttccaggagcg and Rev-cctgcttcaccaccttcttg. The confirmed clones for each construct were chosen based on the best morphology of the pluripotent colonies. One clone per construction was analyzed: *GR* (*G2*), *HGR* (*H5*), *MGR* (*M5*) and *HGR*+*MGR* (*H+M 20*).

### 4.6. Neural Crest Differentiation

mESCs were harvested with Triple Xpress (Gibco), resuspended in fresh DMEM without phenol red LIF or 2i, counted, plated at 2.5 × 10^3^ cells/mL in 24 well-plates with mitotically inactivated PA6 cells for 8 days in differentiation medium (DMEM without phenol red, 10% charcoal-stripped FBS, 2 mM Glutamax, 0.1 mM nonessential amino acids, 1 mM sodium pyruvate and 0.1 mM 2-mercaptoethanol) [6]. According to this protocol, 0.5 nM BMP4 was added from day 5 to day 8 of differentiation and cells were replated on fibronectin-coated dishes in medium containing chick embryo extract to promote neural and smooth muscle differentiation [6,56]. We evaluated the expression of NC, neuronal and smooth muscle cell markers via RT-PCR with specific primers and by immunofluorescence with specific antibodies. 

### 4.7. Immunofluorescence Staining

NIH-3T3 fibroblasts were plated on gelatin-coated coverslips, washed three times in PBS, fixed with 4% paraformaldehyde solution for 10 min, washed again three times with PBS and permeabilized with 0.5% Triton X-100 for 1 h. After blocking for 1.5 h with 10% normal goat serum, samples were probed with primary monoclonal mouse anti-His tag (1:400, ab18184, Abcam, Cambridge, UK) or monoclonal mouse anti-MSX-1/2 (1:500, clone 4G1, Developmental Studies Hybridoma Bank, University of Iowa, IA, USA) antibody at 4 °C overnight, followed by a wash and incubation with goat anti-mouse secondary antibody conjugated with Alexa 488 (1:1000, A-11001, Thermo Fisher Scientific) according to the manufacturer’s instructions. After staining, the percentage of cells with nuclear staining was quantified, either as corrected cell fluorescence (CCF) in the nucleus for the anti-His tag or cells positive for MSX-1/2 in the nucleus.

mESCs were stained with specific antibodies recognizing pluripotent transcription factors, namely, monoclonal mouse anti-Oct4 (1:750, 611202, BD Biosciences, Franklin Lakes, NJ, USA), polyclonal rabbit-anti Sox2 (1:750, AB5603, Millipore Co., Hayward, CA, USA) and polyclonal rabbit anti-Nanog (1:1000, 500-P236, Pepro Tech, Inc. Rocky Hill, NJ, USA) antibodies. Resistant mESC clones were stimulated with Dex and assessed for neural markers using polyclonal rabbit anti-Tuj1 (anti-β-Tubulin III, 1:1000, MRB-435P-100, BioLegend Inc, San Diego, CA, USA), monoclonal mouse anti-α-Smooth Muscle Actin (1:400, clone1A4 A2547, Sigma-Aldrich Milwaukee, WI, USA) and monoclonal mouse anti-Col2a1 (1:200, sc-518017) antibodies. Secondary goat anti-mouse IgG (H+L) Alexa-fluor 488 (1:1000, A-11001, Thermo Fisher Scientific, Waltham, MA, USA) and goat anti-rabbit IgG (H+L) Alexa-fluor 568 (1:1000, A-11011, Thermo Fisher Scientific) antibodies were used. 

### 4.8. RNA Extraction and RT-PCR

Total RNA was isolated from pluripotent and differentiated cells using TRIzol reagent (Molecular Research Center Inc, Cincinnati, OH, USA). Then, 1 μg of RNA was reverse transcribed using 10 mM dNTPs (Promega, Madison, WI, USA), 100 U of Superscript III Reverse Transcriptase, and 500 ng of oligo (dT) 12-18 (Invitrogen, Carlsbad, CA, USA) to produce cDNA. PCR reactions were performed using 300 ng of cDNA, 2 U of recombinant *Taq* DNA polymerase (Invitrogen), 20 pmol of each oligonucleotide, dNTPs (Promega, Madison, WI, USA), 1.5 mM MgCl_2_ (Invitrogen, Carlsbad, CA, USA) and 0.5 μM of the following specific primers. For *Hes1* (NM_008235): Fw-tctacaccagcaacagtgg and Rev-tcaaacatctttggcatcac; *Msx1* (NM_010835.2): Fw-cctgaaaccaggcaggactt and Rev-ggatgcttgagagccacgaa; *Snai1* (NM_011427): Fw-gtggaaaggccttctctaggc and Rev-cagactcttggtgcttgtgg; *Sox9* (NM_011448): Fw-agctcaccagaccctgagaa and Rev-ccagcaatcgttaccttc; *Sox10* (NM_011437): Fw-gcaggaagggttagggtagg and Rev-gcggagaaaggatcagagtg. PCR products were analyzed via electrophoresis to determine their molecular weights after ethidium bromide staining. Reactions with RNA in the absence of retro-transcription were included as negative controls. The housekeeping genes β-Actin (NM_007393) (Primers: Fw-gggtcagaaggattcctatg and Rev-ggtctcaaacatgatctggg) and *Gapdh* (NM_001289726.1) (Primers: Fw-atcaccatcttccaggagcg and Rev-cctgcttcaccaccttcttg) were used to normalize the gene expression levels. The expression of neural and NC markers was evaluated in GR, HGR, MGR, and HGR+MGR cell lines after induction of nuclear translocation by Dex, and the results were compared to the condition without Dex.

### 4.9. qPCR

Real-time polymerase chain reaction (PCR) was carried out using iTaq Universal SYBR Green Supermix (Bio-Rad, Hercules, CA, USA) and a CFX96 Real-Time System (Bio-Rad, Hercules, CA, USA). The primer concentration was 0.3 μM. Forty reaction cycles were performed followed by melt curves to confirm the specificity of the primer sets. Three biological replicates were measured. We used the following specific primers. For *α-Sma/Acta2* (NM_007392.3): Fw-ctaaggccaaccgggagaaa and Rev-agtccagcacaataccagttgt; *β-Tubulin III* (NM_023279.2): Fw-ggcctcctctcacaagtatgt and Rev-ttgccagcaccactctgac; *Col2a1* (NM_001113515.2): Fw-gaaggtgctcaaggttctcg and Rev-ctccaggaataccatcagtcc; *Nanog* (NM_028016.3): Fw-tatctggtgaacgcatctgg and Rev-ctgctccgctccataacttc; *Oct4* (NM_001252452.1): Fw-gggctagagaaggatgtggtt and Rev-ggaaaagggactgagtagagtgtgg; *Pax6* (NM_001244198.2): Fw-caccagactcacctgacacc and Rev-cgaatacggggctctgagaa; Sox1 (NM_009233.3): Fw-atgatgcaggaggcacagc and Rev-ccatgcaccgctacgacat; *Sox2* (NM_011443.4): Fw-gcacatgaacggctggagcaacg and Rev-tgctgcgagtaggacatgctgtagg; *Col2a1* (NM_001113515.2) Fw-gaaggtgctcaaggttctcg and Rev-ctccaggaataccatcagtcc; *Col10a1* (NM_009925.4) Fw-caatggcagcagcattacg Rev-gcgtgccgttcttatacagg; *Aggrecan* (NM_007424.3) Fw-ccttctccagcttcttgagc and Rev-ggcagtggacttgtctcagg; for normalization, we used the housekeeping gene *β-Actin* (NM_007393): Fw-gggtcagaaggattcctatg and Rev-ggtctcaaacatgatctggg.

### 4.10. Micromass Culture

After 8 days of differentiation in stromal PA6 cells, cells were harvested and counted. Micromass cultures were obtained by pipetting a 10 μL droplet (2 × 10^5^ cells) of cell suspension in 24-well plates. Following a 3 h attachment period without medium, differentiation medium was gently added, and changed every other day. The day of plating in micromass culture was designated as day 0. Starting on day 1, the culture medium was changed, and dexamethasone or vehicle was added, as indicated in Figure 1. After 3 or 10 days, micromass cultures were rinsed twice with PBS and fixed with Khale solution for 20 min at room temperature. This solution was substituted by 0.5% Alcian blue solution in 0.1 N HCl. After two days of staining, cultures were washed two times with 0.1 N HCl for 5 min. The cultures were stored in PBS until microphotography analysis [67]. The images of each well were digitalized by a color scanner (Hp Deskjet Ink Advantage 2135, Palo Alto, CA, USA), and are shown as insets for each condition. The microscopic pictures were acquired with an inverted microscope (Nikon DIAPHOT, Minato, Tokio, Japan) and digitalized to 8 bits. These images were further processed to subtract background and to add the “Cyan hot” filter, using the open access program Fiji (Figure 10 A–H). Higher magnifications images were acquired with a Nikon eclipse TE2000-U inverted microscope and minimally adjusted for brightness and contrast (Figure 10 I–X).

### 4.11. Statistical Analysis

Experiments were performed in triplicate with at least 3 independent replicates. The results are expressed as the mean ± SEM. One-way or two-way ANOVA was applied followed by Tukey’s multiple comparison test. *p* values ≤0.05 were considered statistically significant.

## 5. Conclusions

Altogether, our results show that the activation of two key genes that have been demonstrated to be pivotal in the development of multiple lineages in chick, mouse, and frog embryos has an effect on differentiation of the generated mESC lines into NC cells. Most protocols for NC differentiation of ESCs require several exogenous cell signaling molecules to facilitate differentiation into NC-derived cells [68]. Remarkably, in this work, the lineages obtained were differentiated through activation of the HES-1 and MSX-1 transcription factors in addition to the extracellular stimulation. It is tempting to speculate that these differentiated α-SMA-expressing cells will provide a valuable in vitro model to gain insights into the genes and pathways involved in the formation of smooth muscle cells for research in vascular development. Vascular smooth cells are neural crest derivatives, a cell population which is important in physiological and pathological processes that are not yet completely understood (i.e., the neurocristopathies CADASIL and Alagille syndromes) [1]. On the other hand, MGR cells might be used to produce chondrocytes at higher efficiencies, another NC-derived cell type which is highly relevant for craniofacial development

## Figures and Tables

**Table 1:** **Plasmid List.** Gene sequences can be found on the NCBI GenBank database.

Plasmid Name	NCBI Accession Number and Name	Vector Backbone	Reference
hGR-pcDNA3.1-myc-His	HQ692822, *Homo sapiens glucocorticoid nuclear receptor* mRNA	pcDNA3.1(+)/myc-His A™ Invitrogen	Current work
*mHes1*-hGR-pcDNA3.1-*myc*-His	BC051428, *Mus musculus hairy and enhancer of split 1*, mRNA	hGR-pcDNA3.1-myc-His (Current work)	Current work
*mMsx1*-hGR-pcDNA3.1-Hygro	BC016426, *Mus musculus homeobox 1, msh-like 1*, mRNA	hGR-pcDNA3.1-myc-His (Current work)	Current work

## Supplementary Materials

Supplementary materials can be found at http://www.mdpi.com/1422-0067/19/12/4025/s1.

## Abbreviations

BMP	Bone morphogenetic protein
COL2A	Collagen type II alpha 1 chain
Dex	Dexamethasone
ESC	Embryonic stem cells
FBS	Fetal bovine serum
FGF	Fibroblast growth factor
GR	Glucocorticoid binding domain
HES-1	Hairy and enhancer of split 1
HGR	Hes1-GR
LIF	Leukemia inhibitory factor
MSX-1	Muscle-segment homeobox 1-like
MGR	Msx1-GR
NC	Neural crest
NIH-3T3	Mouse embryo fibroblast cell line
α-SMA	α-Smooth muscle actin
