# Supplementary material for: Activation of Hes1 and Msx1 in Transgenic Mouse Embryonic Stem Cells Increases Differentiation into Neural Crest Derivatives
Source: Int J Mol Sci. 2018 Dec 13;19(12):4025. doi: 10.3390/ijms19124025 (PMC6321090; doi:10.3390/ijms19124025)
Supplement: Supplementary file 1 [file ijms-19-04025-s001.pdf]

Supplementary information

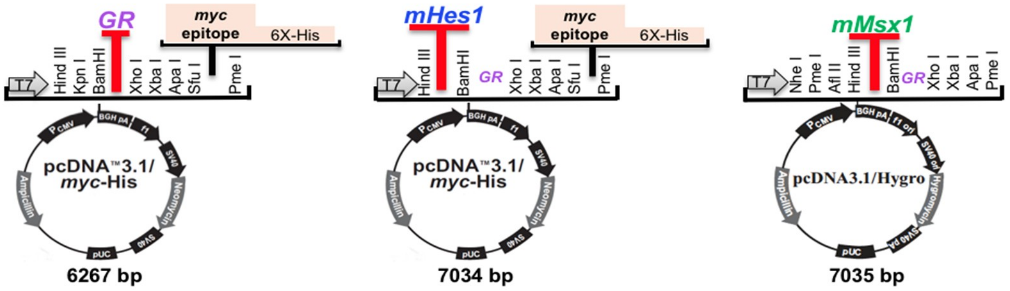

Figure S1. Inducible GR, *Hes1* and *Msx1* DNA constructs. Plasmid maps depicting the insertion of GR (6267 bp) into pcDNA3.1; this vector was used to clone in-frame either *Hes1* (7034 bp) or *Msx1* (7035 bp).

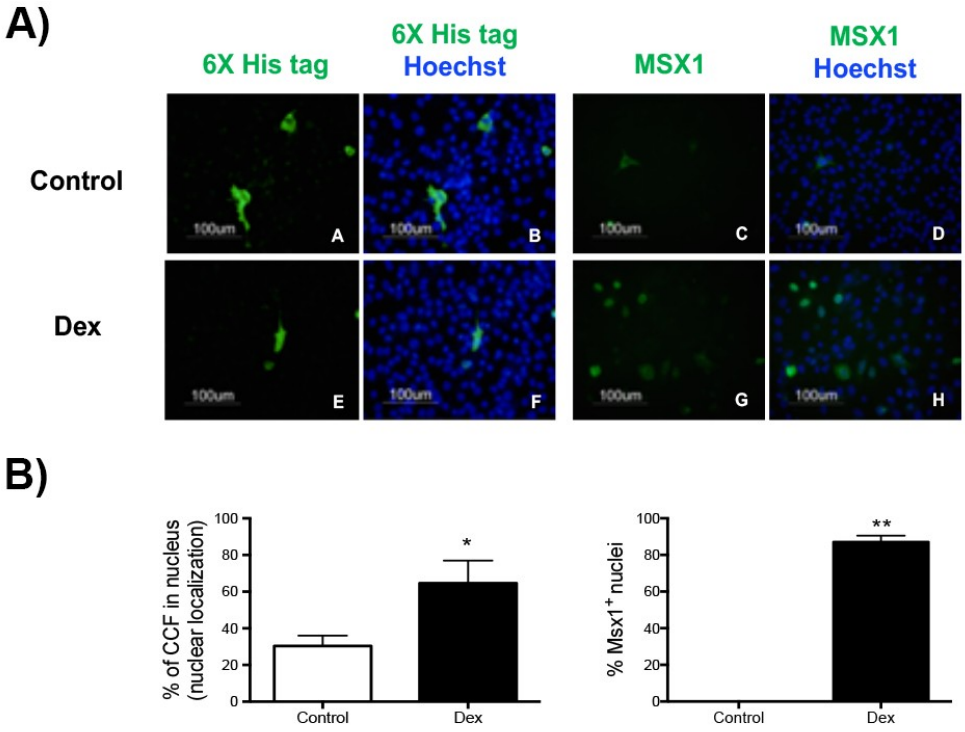

Figure S2. Transient transfection of mouse fibroblasts to verify nuclear translocation after dexamethasone addition. **(A)** NIH-3T3 fibroblasts were transfected with *HGR* (left side) or *MGR* (right side) for 48 hours. Afterwards, cells were treated with 100 µM dexamethasone, and nuclear translocation was evaluated after 24 hours using a specific 6X-His tag or Msx1 antibody. **(B)** Quantification (using ImageJ) of corrected cell fluorescence of 6X-His tag staining in the nucleus of untreated cells or cells stimulated with dexamethasone. The percentage of Msx1-positive nuclei clearly increased after dexamethasone addition. Statistical analysis, Student's t-test; *p< 0.05, and **p<0.01.
